# Healthcare Professionals' Attitudes Toward Patients With Mental Illness: A Cross-Sectional Study in Qatar

**DOI:** 10.3389/fpsyt.2022.884947

**Published:** 2022-05-16

**Authors:** Suhaila Ghuloum, Ziyad R. Mahfoud, Hassen Al-Amin, Tamara Marji, Vahe Kehyayan

**Affiliations:** ^1^Hamad Medical Corporation, Doha, Qatar; ^2^Division of Epidemiology, Department of Population Health Sciences, Weill Cornell Medicine, Cornell University, New York, NY, United States; ^3^Department of Medical Education, Weill Cornell Medicine-Qatar, Al-Rayyan, Qatar; ^4^Primary Health Care Corporation, Doha, Qatar; ^5^Faculty of Nursing, University of Calgary in Qatar, Doha, Qatar

**Keywords:** mental illness, stigma, healthcare professionals (HCPs), attitude, Qatar

## Abstract

**Background:**

Negative attitudes toward mental illness by Health Care Professionals (HCP) have been reported in many countries across the world. Stigmatizing attitudes by HCP can have adverse consequences on people with mental illness from delays in seeking help to decreased quality of care provided. Assessing such attitudes is an essential step in understanding such stigma and, if needed, developing and testing appropriate and culturally adapted interventions to reduce it.

**Aims:**

To assess physicians and nurses attitudes toward mental illness and to determine associated factors with different levels of stigma.

**Methods:**

A cross-sectional survey was conducted among Physicians and Nurses. The Mental Illness Clinician's Attitudes (MICA) scale was used to assess attitudes toward mental illness. MICA scores range between 1 and 6 with higher values indicating higher stigmatizing attitudes. Demographic and work related information were also gathered. Descriptive statistics along with multivariate linear and multivariate logistic regression models were used.

**Results:**

A total of 406 nurses and 92 doctors participated in the study. The nurses' mean MICA score was significantly higher than that of the physicians. Among nurses, being Asian and working in a geriatric, rehabilitation and long-term care facility were associated with lower MICA scores. Among physicians, being female or graduating more than 1 year ago were also associated with lower MICA scores.

**Conclusion:**

Stigmatizing attitudes toward people with mental illness by healthcare workers are present in Qatar. They are higher among nurses as compared to physicians. Factors associated with higher stigmatizing attitudes could be used in creating appropriate intervention to reduce the magnitude of the problem.

## Introduction

Stigmatizing attitudes toward persons with mental illness (PWMI) is widespread in populations globally (1–5). It is also evident in Arab cultures (6–10). Population studies have shown that the public considers PWMI dangerous and mentally retarded (11) or possessed by evil spirits (8, 12, 13). Some Muslim families attribute mental illness to being tested or punished by God (8). PWMI internalize such stigmatizing stereotypes with negative consequences on several aspects of their lives, such as seeking gainful employment, education, or close relationships (14, 15). Aside from self-perceptions of being devalued because of their mental illness, stigma related to mental illness has also been a major barrier to accessing mental health services and receiving timely treatment (16–18) with about two-thirds of the population with mental disorders not receiving treatment (19). Untreated mental disorders contribute to the burden of disease (20) and are “associated with risk factors for chronic disease such as smoking, reduced activity, poor diet, obesity, and hypertension” (21).

Healthcare professionals (HCP) also share the public's stigmatizing attitudes toward PWMI (22–24). Patients have pointed out mental health care professionals as the most stigmatizing (17, 22). Such stigmatizing attitudes may influence the quality of their relationship and care provision toward PWMI (17, 24, 25) and thus impede the help-seeking behaviors in PWMI (26). Their stigmatizing attitudes may also lead to longer waiting times, sub-standard care, verbal or physical abuse (27), and poor prognosis (28). Healthcare professionals' limited experience with and knowledge of mental disorders contributes to their negative attitudes (29). As physicians are often at the forefront of a health care system as practitioners and educators, their stigmatizing attitudes may also influence other members of the team and future practitioners (30, 31).

Most of the published research focused on public attitudes and perceptions toward mental illness and those with lived experience, but the extent of stigma among HCPs toward the same population is less studied (22) and the findings are also mixed. A study in the United States that compared the attitude of mental health professionals and the general public showed more positive attitudes in mental health professionals (31). Another study of nurses' attitudes in Finland showed positive attitudes (32). Ar study from Palestine with mental health professionals showed positive and negative attitudes toward PWMI (33). A survey of medical students in Qatar showed that many students perceived mental illness as a punishment from God, that PWMI should not get married, and that they would be ashamed to have a family member with mental illness (34). Studies in Jordan and Kuwait affirmed the stigmatizing attitudes of mental health professionals (28, 35). Recent studies in Saudi Arabia and Jordan revealed high stigmatizing attitudes among tertiary hospital physicians and health care providers, respectively, toward people with mental disorders (36, 37).

Thus, stigmatizing attitudes have negative consequences in PWMI and the studies with HCPs are few and not conclusive especially in Middle Eastern countries (38) and the Arabic Peninsula. We conducted these attitudinal studies in HCPs in the cosmopolitan population of Qatar, one of the rapidly growing countries in the Middle Eastern North African (MENA) region. The primary aim of this study was to examine their attitude toward mental illness and PWMI. A secondary aim was to explore the associations between the variable attitudes and their psychosocial and professional characteristics.

## Methods

### Design and Setting

This was a descriptive cross-sectional study to explore the attitudes of nurses and physicians working at Hamad Medical Corporation (HMC) in Doha, Qatar toward people with mental illness, and to examine the demographic and professional factors associated with different levels of stigma. HMC is the largest healthcare provider in the State of Qatar, and the main provider of secondary and tertiary care. It consists of 12 general and specialized hospitals as well as ambulatory and community care facilities. The corporation employs tens of thousands of staff, representing more than 70 different nationalities (39). Nurses, followed by physicians, represent the vast majority of clinical staff, and come from different cultural, academic, and training backgrounds. The following services at HMC were selected as they commonly interact with PWMI: Emergency, Medical, and Mental Health Services.

### Ethical Clearance

Approvals were obtained from the Institutional Review Boards (IRB) of HMC (16231/16), Weill Cornell Medicine—Qatar (16-00016), and University of Calgary, Canada (REB16-0878_MOD3). The study was funded by a grant from Qatar National Research Fund, National Priorities Research Program (NPRP No. 9-270-3-050). As the study was done online, waiver of informed consent was approved through the ethics review boards. Acceptance to participate in the survey was considered as a consent.

### Participants' Recruitment and Data Collection

Information letters were emailed to members of the senior management team of the study settings. Meetings were conducted with the chairs of the relevant departments and nurse leaders to promote the study and encourage active engagement. An information sheet for potential participants in the study was prepared along with an invitation letter encouraging clinicians to participate. The final study instrument included the MICA scale and a background questionnaire covering sociodemographic, training, and experience measures. Data collection was done online from April 2017 to February 2018 using SurveyGizmo. All clinicians in HMC use English as the main language of communication and documentation and thus the surveys were conducted in English. All data were collected anonymously. Several reminder emails were sent to promote survey participation.

### Measure of Attitude Toward Mental Illness

The Mental Illness Clinician's Attitude Scale (MICA-4) was used to assess healthcare professionals' (registered nurses and physicians) attitude toward mental illness (40, 41). MICA is a 16-item self-administered questionnaire. It has been validated for use with professionals working in the mental health field as well as other professionals (40). It covers five factors: views of healthcare and mental illness, knowledge of mental illness and patient care, distinguishing physical and mental healthcare, and disclosure. Items are scored on a 6-point Likert scale ranging from 1 (strongly agree) to 6 (strongly disagree). Some of the items are reverse coded. Participant's MICA score is their average score on the 16 items with higher scores indicating higher stigma.

### Statistical and Data Analysis

#### Sample Size Calculations

With 100 physicians and 400 nurses the study will be able to estimate the mean value of the MICA scale to within <20% of a standard deviation value among the physicians and 10% of a standard deviation value among the nurses, respectively, using a 95% confidence interval.

Demographics and work-related variables were summarized using frequency distributions. Simple and multivariate linear regression models were fitted to assess the association between the MICA score (dependent variable) and the demographic and profession-related variables (independent measures). Analyses were done using IBM-SPSS (version 26, Armonk, NY, USA). Significance level was set at the 5% level.

## Results

### Background Characteristics of Participants

The total number of participants who completed the online MICA was 406 nurses and 92 physicians. Table 1 shows the sociodemographic background data of the respondents. Female nurses made up 56.7% (*n* = 229) and female physicians 35.9% (*n* = 33) of the samples. The majority of the physicians were Arab nationals (64.2%) and that of nursing staff were Asians (40.6%) and South Asians (33%). only 21.4% of the nursing staff were of Arab ethnicity. The majority of physicians (93.5%) were Muslim, as opposed to only 28.3% of nurses; 65.0% of nurses were Christian.

**Table 1:** Participants' sociodemographic background characteristics.

**Variable**		**RN**		**MD**	
		***N***	**%**	***N***	**%**
Gender	Male	175	43.3%	59	64.1%
	Female	229	56.7%	33	35.9%
Ethnicity	Qatari Arab	3	0.7%	11	12.0%
	GCC Arab	3	0.7%	1	1.1%
	Other Arab	81	20.0%	47	51.1%
	Asian (Philippine Thailand Chinese Korean)	165	40.6%	2	2.2%
	South Asian (Indian Bangladesh Nepal Pakistani, Sri Lanka)	134	33.0%	18	19.6%
	African	12	3.0%	9	9.8%
	Other ethnicities	3	0.7%	3	3.3%
	Not disclosed	5	1.2%	1	1.1%
Household income per month (QR)	5,000–10,000	259	64.1%	2	2.2%
	10,001–20,000	114	28.2%	26	28.3%
	20,001–30,000	6	1.5%	21	22.8%
	30,001–40,000	3	0.7%	7	7.6%
	40,001–50,000	1	0.2%	6	6.5%
	50,001 and over	1	0.2%	26	28.3%
	Not disclosed	20	5.0%	4	4.3%
Religion	Muslim	115	28.3%	86	93.5%
	Christian	264	65.0%	4	4.3%
	Hindu	14	3.4%	0	0.0%
	Other	9	2.2%	0	0.0%
	Not disclosed	4	1.0%	2	2.2%

*RN, Registered Nurse; MD, Medical Doctor*.

As shown in Table 2, the educational level for most of the nurses was a bachelor's degree (78.0%), and a minority (4.4%) had higher education. Most nurses (70.8%) had seven or more years' experience since graduation, and 50.0% worked for HMC for a similar period. Among physicians, 38.5% had seven or more years' experience since graduation, and 25.6% had worked either 1–2 or 7 years or more in HMC. Most physicians (77.8%) and nurses (72.3%) reported contact with a person with mental illness in a non-professional capacity. For nurses, 41% reported daily contact with PWMI; for physicians, the distribution was quite uniform for daily, weekly, or monthly contact.

**Table 2:** Participants' professional background characteristics.

**Variable**		**RN**		**MD**	
		***N***	**%**	***N***	**%**
MD grade	Resident			50	57.5%
	Fellow Jr			5	5.7%
	Fellow Sr			4	4.6%
	Consultant			25	28.7%
	Not disclosed			2	2.3%
	Other			1	1.1%
Level of nursing education	Diploma	71	17.5%		
	Bachelor's degree	316	78.0%		
	Master's degree	18	4.4%		
Department/unit employed	Medicine	131	32.5%	34	37.0%
	Emergency department	78	19.4%	29	31.5%
	Neurology department	9	2.2%	0	0.0%
	Hamad General Hospital Outpatient Clinic	1	0.2%	0	0.0%
	Rumailah hospital	141	35.0%	26	28.3%
	Other	43	10.7%	3	3.3%
Years since graduating	<1	7	1.8%	3	3.3%
	1–2	6	1.5%	19	20.9%
	3–4	43	10.8%	22	24.2%
	5–6	61	15.3%	12	13.2%
	7 and over	283	70.8%	35	38.5%
Length of service (in years)	<1	49	12.3%	15	16.7%
	1–2	39	9.8%	23	25.6%
	3–4	62	15.5%	17	18.9%
	5–6	47	11.8%	11	12.2%
	7 and over	200	50.0%	23	25.6%
	Not disclosed	3	0.8%	1	1.1%
Personal experience or contact (non-professional capacity) with a person with mental disorder	Yes	290	72.3%	70	77.8%
	No	105	26.2%	18	20.0%
	Not disclosed	6	1.5%	2	2.2%
Professional experience or contact (while providing care) with a person with mental disorder	Never	29	7.3%	7	7.8%
	Daily basis	164	41.0%	31	34.4%
	Weekly basis	47	11.8%	24	26.7%
	Monthly basis	92	23.0%	24	26.7%
	Yearly basis	68	17.0%	4	4.4%

*RN, Registered Nurse; MD, Medical Doctor; Jr, Junior; Sr, Senior*.

### Association of MICA Scores With Healthcare Professionals' Characteristics

The nurses' overall mean MICA score was 2.87 (SD 0.61; range 1.31–4.94), 95% CI: 2.81–2.93, significantly higher (*p* < 0.001) than that of the physicians' which was 2.55 (SD 0.62; range 1.30–4.13), 95% CI: 2.42–2.68. The MICA scale's internal consistency and reliability among nurses and medical physicians were reasonable, with values of Cronbach alpha 0.729 for nurses and 0.768 for medical physicians. Tables 3, 4 describe the associations between the MICA scores with the nurses' and physicians' characteristics. Among nurses, at the bivariate level, the MICA scores were significantly associated with ethnicity, education, services where they work, personal experience with PWMI, and the frequency of taking care of PWMI. At the multivariate level, the MICA scores remained significantly associated with ethnicity and department of work. In particular, Asian nurses had a significantly lower MICA score than Arab nurses (adjusted mean diff = 0.426, *p* < 0.001), and those who work in the Emergency Department scored significantly lower than those in Hamad General Hospital (HGH) (adjusted mean diff = 0.336, *p* = 0.004) (See Table 3).

**Table 3:** Association between MICA scores and nurses' characteristics.

	**MICA**			**Unadjusted**			**Adjusted**		
**Variable**		**Mean**	**SD**	**Slope**	**Standard error**	***p*** **-value**	**Slope**	**Standard error**	***p*** **-value**
Gender	Male	2.84	0.64	1					
	Female	2.90	0.59	0.059	0.062	0.342			
Ethnicity	Arab	2.99	0.65	1			1		
	Asian (Philippine Thailand Chinese Korean)	2.69	0.57	−0.302	0.078	<0.001*	−0.426	0.082	<0.001*
	South Asian (Indian Bangladesh Nepal Pakistani, Sri Lanka)	3.05	0.55	0.056	0.082	0.493	−0.153	0.093	0.102
	Other Ethnicities	2.81	0.68	−0.181	0.164	0.271	−0.146	0.162	0.368
Income	5,000–10,000	2.89	0.62	1					
	10,001–20,000	2.83	0.60	−0.059	0.070	0.400			
	Above 20,000	3.06	0.83	0.174	0.190	0.360			
Religion	Muslim	2.93	0.63	1					
	Christian	2.84	0.60	−0.083	0.069	0.230			
	Other	3.00	0.62	0.073	0.145	0.616			
Education	Diploma	2.98	0.67	1			1		
	Bachelor's degree	2.87	0.59	−0.113	0.081	0.163	−0.002	0.089	0.978
	Master's degree	2.55	0.56	−0.432	0.160	0.007*	−0.189	0.161	0.241
Department	Medicine	2.97	0.51	1			1		
	Emergency Department	2.99	0.60	0.013	0.085	0.877	0.016	0.088	0.854
	Mental Health Service	2.64	0.66	−0.330	0.072	<0.001*	−0.336	0.116	0.004*
	Other department Neurology?	3.05	0.55	0.070	0.098	0.471	0.041	0.099	0.675
Years since degree	<1	2.90	0.86	1					
	1–2	3.04	0.84	0.143	0.341	0.674			
	3–4	2.98	0.58	0.082	0.25	0.743			
	5–6	2.85	0.65	−0.044	0.245	0.857			
	7 and over	2.86	0.60	−0.400	0.234	0.866			
Length of service (in years)	<1	2.75	0.54	1					
	1–2	2.92	0.58	0.172	0.132	0.194			
	3–4	2.85	0.57	0.104	0.119	0.383			
	5–6	2.89	0.77	0.142	0.126	0.257			
	7 and over	2.88	0.60	0.128	0.098	0.19			
Personal Experience with people with Mental Health disorders	Yes	2.83	0.59	1			1		
	No	2.98	0.63	0.154	0.070	0.028*	0.052	0.073	0.477
Frequency of taking care of patients with mental disorder	Never	3.10	0.57	1			1		
	Daily basis	2.71	0.65	−0.398	0.120	0.001*	−0.119	0.155	0.441
	Weekly basis	3.00	0.55	−0.102	0.141	0.468	−0.054	0.152	0.721
	Monthly basis	2.95	0.55	−0.157	0.127	0.218	−0.06	0.136	0.659
	Yearly basis	2.96	0.55	−0.143	0.133	0.281	−0.072	0.138	0.605

*ED, Emergency Department; MHS, Mental Health Services; MICA, Mental Illness Clinician Attitude; SD, Standard Deviation*.

* *Signifies significant*.

**Table 4:** Association between MICA scores and physicians' characteristics.

	**MICA**			**Unadjusted**			**Adjusted**		
**Variable**		**Mean**	**SD**	**Slope**	**Standard error**	***p*** **-value**	**Slope**	**Standard error**	***p*** **-value**
Gender	Male	2.70	0.62	1					
	Female	2.28	0.53	−0.42	0.132	0.002*	−0.35	0.145	0.019*
Ethnicity	Arab	2.61	0.67	1					
	Asian	2.54	0.47	−0.068	0.172	0.695			
	Other Ethnicities	2.33	0.52	−0.279	0.205	0.176			
Income	5,000–20,000	2.68	0.56	1			1		
	20,001–30,000	2.75	0.67	0.078	0.176	0.659	0.235	0.204	0.254
	Above 30,000	2.36	0.57	−0.316	0.150	0.039*	0.146	0.225	0.517
Religion	Muslim	2.57	0.61	1					
	Christian	2.23	0.58	−0.332	0.313	0.292			
Education	Residents	2.69	0.54	1			1		
	Fellows	2.62	0.94	−0.066	0.228	0.773	0.07	0.254	0.784
	Consultants	2.33	0.58	−0.363	0.156	0.022*	−0.366	0.327	0.911
Department	Medicine	2.56	0.64	1			1		
	Emergency Department	2.80	0.52	0.25	0.155	0.117	0.167	0.18	0.357
	Mental Health Service	2.23	0.56	−0.330	0.161	0.043*	−0.45	0.417	0.236
	Other department Neurology??	2.77	0.83	0.214	0.354	0.547	0.582	0.486	0.237
Years since degree	<1	3.56	0.80	1			1		
	1–2	2.75	0.54	−0.815	0.444	0.07	−1.116	0.423	0.011*
	3–4	2.61	0.53	−0.954	0.441	0.033*	−1.062	0.41	0.012*
	5–6	2.39	0.60	−1.176	0.459	0.012*	−1.072	0.475	0.028*
	7 and over	2.38	0.66	−1.184	0.436	0.008*	−1.285	0.529	0.018*
Length of service (in years)	<1	2.52	0.38	1					
	1–2	2.73	0.58	0.213	0.208	0.308			
	3–4	2.69	0.81	0.174	0.227	0.444			
	5–6	2.33	0.70	−0.185	0.262	0.481			
	7 and over	2.40	0.61	−0.113	0.21	0.593			
Personal Experience with people with Mental Health disorders	Yes	2.54	0.65	1					
	No	2.61	0.57	0.0661	0.172	0.702			
Frequency of taking care of patients with mental disorder	Never	3.21	0.51	1			1		
	Daily basis	2.30	0.61	−0.911	0.245	<0.001*	−1.76	0.442	0.691
	Weekly basis	2.62	0.73	−0.59	0.249	0.021*	−0.46	0.256	0.078
	Monthly basis	2.51	0.30	−0.696	0.254	0.008*	−0.512	0.259	0.053
	Yearly basis	3.06	0.39	−0.149	0.401	0.710	−0.01	0.404	0.98

*ED, Emergency Department; MHS, Mental Health Services; MICA, Mental Illness Clinician Attitude; SD, Standard Deviation*.

* *Signifies significant*.

Bivariate analysis showed that among physicians, there were significant associations between the MICA score and gender, income, education, type of clinical service, years since degree, and frequency of taking care of patients with a mental disorder. At the multivariate level, only gender and years since degree remained significantly associated with the MICA score. In particular, the average MICA score for female physicians was lower than that of male ones (adjusted mean difference 0.35, *p* = 0.019). Those who graduated more than a year ago scored lower on MICA than those who graduated <1 year ago (see Table 4).

## Discussion

In this study, we explored the level of stigmatizing attitudes toward mental illness among HCPs working in several HMC departments. The sociodemographic findings reflect the workforce composition of HMC, whereby there is reliance on international recruitments to meet the staffing needs of Qatar's healthcare system. The vast majority of nursing staff is recruited from India and the Philippines, followed by North Africa. The knowledge and experience of health staff in their undergraduate and subsequent professional life are quite varied; this is likely to influence their attitude toward patients with mental illness. Studies in different cultures have reported cultural influences on mental illness-related stigma (42, 43). In the Arab world, cultural values influence attitudes and types of treatments sought (6, 8, 10). Burgut and Polan (44) compared the attitudes of medical students in Qatar and New York (44). They reported more negative attitudes in medical students in Qatar compared to those in New York. The higher responses from male physicians and female nurses may reflect the general workforce composition in most HMC hospitals and units where most of HCPs tend to be female nurses.

The nurses' mean MICA score was significantly higher than that of physicians, suggesting a higher degree of negative attitude. This finding is consistent with research exploring attitudes of general hospital staff in Malaysia toward mental illness (45). Further, in Sweden, nurses' attitudes toward patients with mental illness were similar to those of the general public (46). Such findings may suggest a global phenomenon. Cremonini et al. (47) have also indicated that nurses may be influenced by the media portrayal of mental illness as potential danger, unpredictability, violence, and being caused by moral weakness. In our sample, diploma graduate nurses had significantly higher mean MICA scores (2.98), with lower scores obtained as the level of education increased. Those with a master's degree had the least mean MICA score. A study from Lebanon also showed that higher level of education was associated with less stigmatizing attitudes toward people with mental illness (48).

Among the physicians surveyed, number of years since obtaining their medical degree was significantly associated with lower negative attitudes; those who received their degree 7 years or more before the survey had the lowest MICA mean score. Thus, duration of practice might contribute to a better understanding of the causes of mental illness and more appreciation of the rights of people with mental illness. Our findings may reflect the standard of training in mental illness at basic graduate levels. As stated earlier, the majority of clinical staff working in HMC is recruited internationally. Most recruited nurses have little undergraduate training in mental health. The curriculum of medical schools in most of the region has little psychiatry training incorporated. In a literature review by Noblett and Henderson (49), several studies demonstrated that attitudes improved with experience (49).

Place of work was another significant variable in our data. Nurses working in the Mental Health Services had a mean MICA score of 2.64. This score is significantly lower than nursing staff working in the other surveyed clinical services. Among the departments surveyed, nurses in the Emergency Department had the highest stigma according to their mean MICA score of 2.99. Physicians working in the Emergency Department also had the highest mean MICA score of 2.80, whereas those working at the Mental Health Services had a lower mean score of 2.23. The negative attitude of HCPs working in the Emergency Department is problematic because the people who need acute psychiatric care are initially assessed in this department. The attitudes of HCPs can have serious implications on the quality of care delivered to patients (50). In emergency departments, patients' physical complaints may be overlooked and attributed to their mental illness. Such diagnostic overshadowing is a high risk for worsening morbidity and potentially mortality. It is a major barrier to help-seeking behavior and can cause delays in receiving necessary help with subsequent detrimental effects on quality of life and wellbeing (50, 51). A meta-analysis of general hospital healthcare providers' perception of dangerousness (52) concluded that hospital professionals did not differ from the general public in their perception of PWMI as dangerous. This perception was attributed largely to staff feeling inadequately trained and thus unprepared to managing PWMI.

Our findings of lower stigma among mental health professionals are consistent with a literature review that concluded that these professionals had fewer negative attitudes toward mental illness than non-mental health professionals (49) but contradict those by Ahmead et al. (33). The latter found that mental health professionals had a negative attitude toward patients with mental illness. Their conclusion may be explained by the reliance in Palestine on inpatient long-term psychiatric hospital admissions in the absence of community or rehabilitation facilities. In Qatar, the Mental Health Services have a growing community mental health component with daycare and outreach services. The better attitude in these settings is consistent with findings by Cremonini et al. (47) and Arvaniti et al. (53), who refer to the “contact hypothesis”, which stipulates that having professional or personal contact with PWMI results in a more positive attitude (47, 53). They demonstrated variation within mental health settings whereby daycare mental health professionals had a more positive attitude than those in inpatient units. Similarly, staff in inpatient settings had more negative attitudes than those working in outpatient clinics (22). In our study, we did not explore such a degree of variation within our Mental Health Services. Arvaniti et al. (53) investigated the concept of “familiarity” being associated with less social discrimination and more positive attitudes. They found that providers with more contact with patients had more favorable attitudes.

While our data revealed no gender differences in attitude among nurses, the female physicians had a significantly lower stigmatizing attitude toward PWMI than the male ones. Literature evidence on the impact of gender on attitude toward PWMI yields variable results. A study exploring the attitude of Jordanian mental health nurses toward PWMI reported a more positive attitude by female nurses (28). In this study, receiving specialized training in psychiatry had a larger influence on attitude than the level of education. However, other international studies showed no gender differences (22, 54).

The significantly lower stigma among Asian nurses may reflect the cultural beliefs related to the cause of mental illness. In the Arab World, mental illness is largely attributed to reduced faith, not practicing religion, evil eyes, and possession by evil spirits (55–57). Despite education, such beliefs remain largely prominent and can impact not only the healthcare practitioners' understanding of mental illness and its management and prognosis, but also the patient and family's help-seeking behaviors. It is usual for patients with severe mental illness to present late to clinical services after exploring different traditional healing options and failing. This late presentation for help means patients often show in an acutely disturbed mental health state that may be more challenging to treat and may potentially influence healthcare professionals' response.

### Study Strengths and Limitations

This study is the first of its kind in Qatar, with results shedding light on a very important public health issue. The participants in both groups were diverse in demographics and socio-economic levels, which allows for an intriguing exploration of differences in attitude. The use of a validated tool to measure stigma yielded accurate measures of attitude toward mental illness. However, the study also has its limitations. The nurses and physicians who participated in this study might not represent their respective populations, which might limit the generalizability of the results. The small sample size among the physicians did not provide the needed power for some associations with clear trends to reach statistical significance.

## Conclusions

Stigmatizing attitudes toward mental illness by healthcare professionals are significant in Qatar. This study revealed more stigmatizing attitudes among nurses than physicians various healthcare services. Overall, negative attitudes are extant and are likely to affect patient care. Experience and education are the main factors affecting such attitudes. The higher the level of education, the more years of experience, and the more contact healthcare professionals have with PWMI, the better their attitude toward mental illness. These findings will help educators focus their training efforts among healthcare professionals to adopt more positive attitudes toward mental illness. An intervention such as the “contact-based education” that was piloted in China could be assesses for suitability and proposed for its adoption once culturally adapted and tested for reducing stigma among HCPs (58). Factors associated with higher stigmatizing attitudes could also guide policymakers to design better plans to reduce the magnitude of the problem in clinical services.

## Data Availability Statement

The datasets presented in this article are not readily available because after being de-identified and in compliance with the policies and procedures of all universities involved and HMC and Qatar National Research Fund, the datasets generated and/or analyzed during the current study are available from the corresponding author on reasonable request. Requests to access the datasets should be directed to vkehyaya@ucalgary.ca.

## Ethics Statement

Ethical approvals were obtained from the Institutional Review Boards (IRB) of HMC (16231/16), Weill Cornell Medicine—Qatar (16-00016), and University of Calgary, Canada (REB16-0878_MOD3. The Ethics Committee waived the requirement of written informed consent for participation.

## Author Contributions

VK was the principal investigator of this study, was involved in designing the study, data collection, data analysis, drafting, and critically reviewing the manuscript with input from SG, HA-A, and ZM. ZM was involved in conducting the statistical analysis, writing up the statistical analysis, and results sections of the manuscript. SG drafted the manuscript with the rest of authors reviewing and editing it. TM was the lead in data collection, data entry and ensuring data quality, and preparing the data presentation in the manuscript. All authors read and approved the final manuscript.

## Funding

This study was made possible by NPRP No. 9-270-3-050 Grant from the Qatar National Research Fund (a member of Qatar Foundation). The funding agency for this study did not interfere in the study and in the preparation of this manuscript. Open Access funding was provided by the University of Calgary in Qatar.

## Conflict of Interest

SG was employed by Hamad Medical Corporation. The remaining authors declare that the research was conducted in the absence of any commercial or financial relationships that could be construed as a potential conflict of interest.

## Publisher's Note

All claims expressed in this article are solely those of the authors and do not necessarily represent those of their affiliated organizations, or those of the publisher, the editors and the reviewers. Any product that may be evaluated in this article, or claim that may be made by its manufacturer, is not guaranteed or endorsed by the publisher.
